# Experimental and numerical investigation on failure mechanism of expansive soil subgrade slope

**DOI:** 10.1038/s41598-023-46727-3

**Published:** 2023-11-13

**Authors:** Hongri Zhang, Jiming Yang, Lei Wang, Yongfu Xu, Sulian Lan, Junhui Luo, Zhenchao Chang

**Affiliations:** 1 Shanghai Jiao Tong University, Shanghai, 200240 China; 2 Guangxi Transportation Science and Technology Group Co., Ltd, Nanning, 530029 China; 3 Shanghai University of Engineering Science, Shanghai, 201620 China; 4 Guangxi Transport Vocational and Technical College, Nanning, 530023 China; 5 Guangxi Beitou Transportation Maintenance Technology Group Co., Ltd., Nanning, 530029 China

**Keywords:** Natural hazards, Environmental impact, Civil engineering

## Abstract

Shallow landslides of expensive soil slopes occur from time to time, and most engineering geological problems are directly or indirectly caused by soil structure cracks. The existence of tensile cracks can significantly affect the hydro-mechanical properties of soils. In this paper, the mechanism of expansive soil landslide formation is explored, and swelling pressures, and drying and wetting cycles are introduced into the discrete element method (DEM), and the landslide process of expansive soils is studied by the numerical simulation mothed. The relationship between the crack development and the instability of expansive soil slopes was investigated. The results show that under the condition of seasonal dry and wet alternation, the cracks of the expansive soil slope gradually develop, the rainwater infiltrates rapidly, the mechanical properties gradually deteriorate, and under the effect of such adverse cycle, the soil gradually softens and the stability decreases. Under the influence of human activities, vehicle loads and other factors, the slope body slides. These findings are helpful for the design and construction of expansive soil slopes and foundations.

## Introduction

As a special catastrophic soil that absorbs water and expands and loses water and shrinks, the surface of swelling soil slope often produces a large scale and obvious fissure network under the external complex alternating wet and dry environment, which provides a natural preferred channel for water infiltration, accelerates the deterioration of the overall mechanical properties of the slope, breeds the fault surface for geological disasters, and leads to repeated occurrences of landslides and collapses during the construction of highway, high-speed railway and other transportation infrastructure. With the continuous promotion of China's transportation strengthening strategy and the construction of new land and sea channels in the west, a large number of highways, municipal roads, high-speed railways and other infrastructures are built in Guangxi region of western China, which inevitably pass through areas with extensive distribution of expansive soils such as Ningming basin and Baise basin, and emerge a large number of engineering problems such as cracking of expansive slopes, landslides, and shear damage of rigid support structures^1–3^.

The destabilization mechanism of expansive soil slopes is very complex, and its destabilization is mainly controlled by intrinsic factors and external environment^4^. The intrinsic factors are soil expansion and contraction response due to changes in soil moisture^5^, development of surface fracture network^6^, and softening of deep soil due to changes in permeability under the action of wet and dry cycles^7^. The external factors refer to the change of water content of expansive soil slopes due to excavation and unloading, and extreme construction conditions, which induce instability^7–9^. Under the influence of external factors, the surface of the fissures on the slopes of expansive soils gradually develops and the structural strength of the soil is damaged^10^, and the water gradually penetrates into the interior of the slope along the fissures, which in turn leads to the extension of the fissures towards the interior of the slope^11^. Under the effect of further rainfall, the suction of unsaturated expansive soil is rapidly reduced, and the stress difference and strength inhomogeneity are formed inside the slope soil body^12^, and when the shear stress in a certain part increases to its shear strength, shear damage occurs in that part, and the soil strength softens under the joint action of self-weight stress, expansion stress and infiltration force, and the slope undergoes instability damage.

Generally, slope cutting can effectively reduce the landslide possibility for ordinary soil slopes, however, even if the slope rate of expansive soil slopes is reduced to 1:6, i.e., gentle expansive soil slopes, there is still a risk of shallow landslides, and practical engineering applications show that many gentle expansive soil slopes with slopes of 8° to 9° have also experienced landslide^13^. There have been many methods on slope stability evaluation, mainly including limit equilibrium method^14^, plastic limit analysis method^15^, finite element method^16^, artificial intelligence method^17^ and so on. However, since most of the existing research on expansive soil slopes are engineering slopes with relatively large slopes, and the relevant research for gentle expansive soil slopes needs to be further enriched.

In this paper, the engineering background is a gentle expansive soil slope in Ningming, Guangxi, China, and its failure characteristics and incentives are explored through site investigation and survey, and the differences in strength parameters of expansive soil at the slope and slide zone are analyzed by combining dry and wet cycle tests. Finally, based on Janbu method, the stability analysis method of expansive soil slope is established considering the coupling effect of swelling force and dry and wet cycles, and the practical conditions are simulated with DEM due to its strength in large deformation simulation^18^.

## Geomorphology and hazard profile

### Overview of site landslides

**Figure 1:**
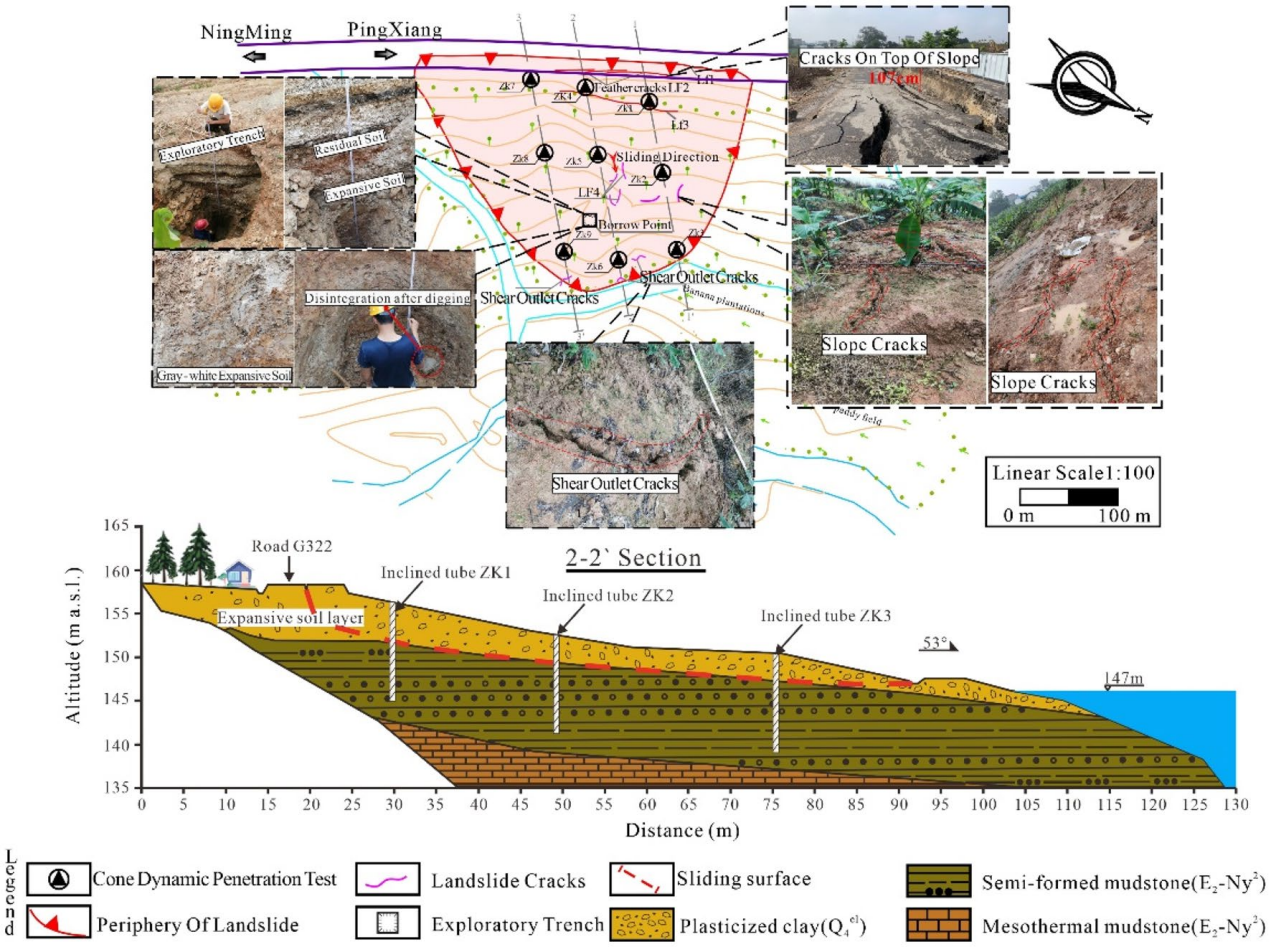
Disaster diagram of expansive soil slope on site.

The overall overview of the study area is shown in Fig. 1. The expansive soil landslide under study is located along a national highway in Guangxi, about 12 km from the city of Ningming County. The study area has a subtropical monsoon climate with an annual rainfall of about 843.3–1439.4 mm, and the rainfall in the flood season from June to October accounts for 81.8% of the annual rainfall. The average monthly sunshine hours are 195.6–212 h, the maximum outdoor temperature is 40.8°, and the average annual evaporation is 1663.7 mm.

Due to the influence of environmental factors such as hot climate, continuous strong rainfall and sun exposure at the site, the expansive soil embankment and pavement cracked in June 2019, and the pavement sank about 20 cm along the fissure under the continuous wet and dry alternation. By March 2020, the pavement fissure developed into multiple feather-shaped fissures, the pavement sank further, and the shear exit fissure at the leading edge of the embankment slope developed significantly, which strongly suggested a potential landslide.

### Destabilization damage state of the expansive soil slope

The results of geological survey on site show that the slope soil layer mainly consists of fill (Q4ml), residual clay (Q4el), and medium to strong weathered mudstone (E2-Ny2), as shown in Fig. 1. The fill is off-white or varicolored, mainly plastic, locally hard plastic, containing plant roots, gravel and debris, The free swelling rate of the fill ranges from 36.0 to 68.0%, with the average value equivalent to 47.9%, and the fill is mainly distributed at the embankment of the back edge of the landslide. The residual clay is generally plastic while locally hard plastic, containing a small amount of weathered rock. Its free swelling rate lies between 24.5 and 70.0%, with an average value of 47.6%. The medium-strong weathered mudstone is dark gray, strongly weathered, with relatively developed joints and fissures. It is softened with increased moisture and shrinks to crack after water loss. The mudstone is colloid and extremely soft, whose free swelling rate is of 10.0% to 60.0% and the average value is 48.5%. It can be seen that the slope soil layer has moderate to weak expansion and contraction capabilities.

**Figure 2:**
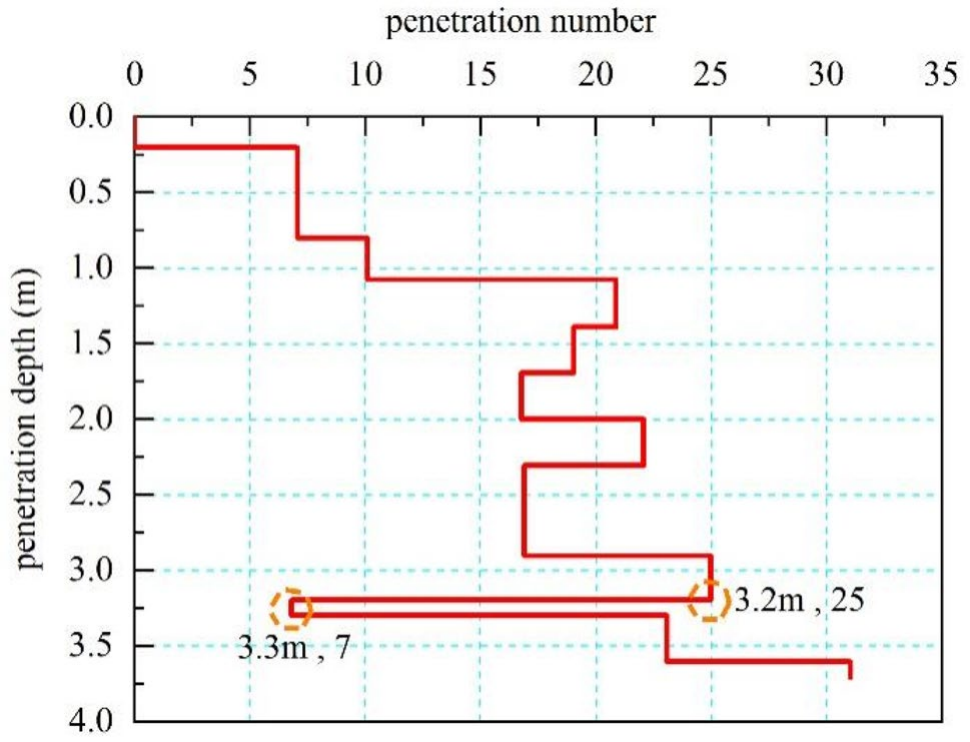
Typical N10-h (penetration strike-depth) curve.

Three inclinometers, numbered ZK1, ZK2 and ZK3, were laid along the 1-1 section of the slope (see Fig. 1). During the survey, it was found that all the inclinometers were sheared at a certain depth. Among them, ZK1 was damaged at the depth of 4.0 m, the ZK2 was damaged at the depth of 3.0 m, and ZK3 was damaged at the depth of 2.7 m. Therefore, the slope was in an unstable state, and a certain degree of sliding deformation was generated leading to the destruction of the inclinometers. The slip surface was located at the location of the inclinometer destruction, and the slip zone was basically located in the expansive clay layer. Meanwhile, the location and shape of the slip surface were further clarified by combining trench excavation and light dynamic penetration test. Figure 1 shows the stratification of the stratum after the excavation of the trench. The light dynamic penetration test can be used to identify the weak interlayer in the stratum, which is a simple survey method. Figure 2 shows the typical N10-h (penetration strike-depth) curve obtained from the light dynamic penetration test. From Fig. 2, it can be seen that the hammering number decreases significantly at the depth of 3.2 m, which indicates that the mechanical properties of the stratum at this depth are poor and hence the stratum is presumed to be at the sliding zone, which basically coincides with the damage depth of the inclinometers mentioned above. The potential slip surface of the slope is shown in Fig. 1, combined with the damage of the inclinometers, the trench excavation and the results of light dynamic penetration test. It can be seen that the slope underwent a shallow destabilization damage, which is also a typical damage mode of expansive soil slope^19, 20^.

As the clay in the landslide body has moderate to weak expansion and contraction, the expansive soil under the influence of climate experiences frequent effects cast by wet and dry alternations, resulting in the development of fissures. The survey results show that fissures with depths ranging from 0.5 to 1.5 m appear at the front edge of the landslide, the middle of the landslide and the back edge of the landslide. When the rainy season comes, the groundwater level rises and the rainwater infiltrates along the fissures to soak the slope soil, making these fissures close or narrow temporarily. Each growth of fissures under the action of a wet and dry cycle intensifies the fissures formed in the previous cycle^21^. In addition, the gradual decrease of soil shear strength (especially cohesion) under the action of dry and wet cycles is the main reason for the damage of expansive soil slopes due to insufficient sliding resistance of the slope to overcome the sliding force^22, 23^. It should be pointed out that expansive clay will also further increase the sliding force after absorbing moisture and swelling and storing water in fissures within the slope, thus aggravating the slope instability damage, which also explains why expansive soil slopes often occur during rainfall.

## Landslide mechanism analysis

### Engineering properties of expansive soils

The physical and mechanical parameters of the in-situ expansive soil at the slope slip zone taken from the field borehole are shown in Table 1. Among them, the cohesive force and internal friction angle of the expansive soil were obtained from the quick shear test. In order to further clarify the correlation between the dry and wet cycling action and the destabilization damage of the expansive soil slope, the dry and wet cycling test of expansive soil was carried out, and the soil samples for the dry and wet cycling test were taken from the slip zone within the slope of the expansive soil slope.

**Table 1:** Main physical and mechanical parameters of expansive soil.

Density (g/cm^3^)	Optimum moisture content (%)	Void ratio	Saturation degree (%)	Specific gravity (g/cm^3^)	Liquid limit (%)
1.82	23	0.996	89.0	2.75	47.9

Plastic limit (%)	Plasticity index	Average value of free swell ratio (%)	Cohesion (kPa)	Internal friction angle (°)	Vertical expansive pressures (kPa)
24.6	23.3	48.5	8.8	9.1	40.8

The expansive soil was crushed, dried, sieved and made into ring knife specimens with 90% compaction and subjected to 0, 2, 4, 6 and 8 dry and wet cycles, respectively. The site investigation showed that the moisture content of the local expansive soil mainly fluctuated in the range of 10% to 34%. Therefore, the upper and lower limits of moisture content in the dry and wet cycles were set at 10% and 35%, respectively. Currently, there is no uniform standard regarding the implementation method of the wet and dry cycle. The combination of water spraying and drying to simulate the wet and dry cycle of soil samples is widely used^24^. In this paper, this approach is also adopted, i.e., the specimen was sprayed with water by a small spray bottle, weighed from time to time, and considered wet when its mass reached the value corresponding to the moisture content of 35%. Then, the wet specimens were baked in an oven at 40° and weighed from time to time, and considered dry when their mass reached the corresponding moisture content of 10%, which completed one dry and wet cycle. This operation was repeated to simulate the actual service environment of the local slope soil.

### Indoor wet and dry cycle test

**Figure 3:**
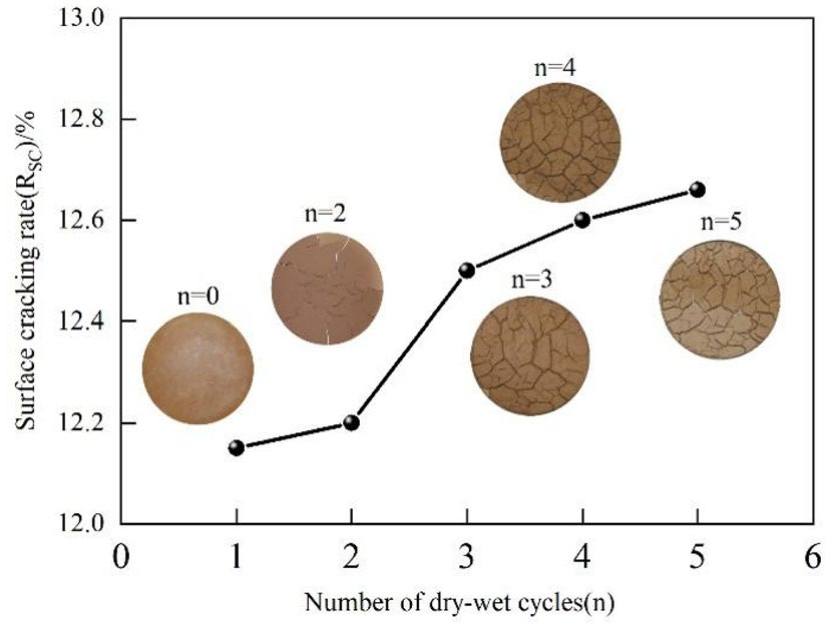
Disaster diagram of expansive soil slope on site.

**Figure 4:**
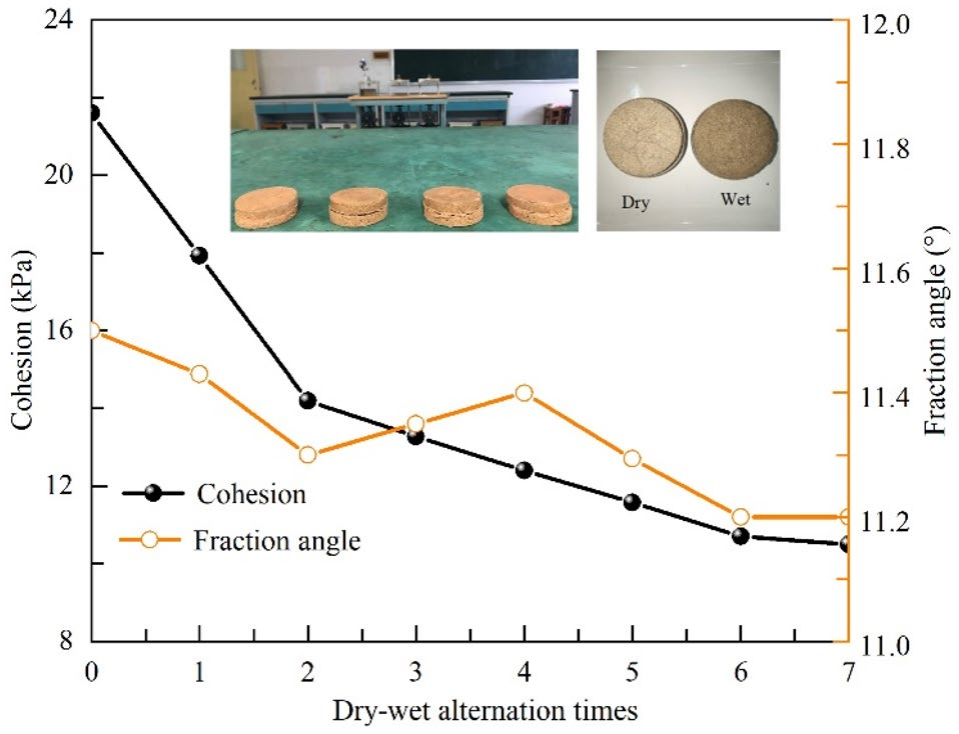
Disaster diagram of expansive soil slope on site.

The environmental conditions of the slope failure site of expansive soil embankment are quite severe, with long-term drought often followed by long-term continuous rainfall, and hence the continuous effect of dry and wet cycles is obvious. In order to explore the deterioration of shear strength of expansive soil slopes under extreme wet and dry cycling conditions, experiments on the fissure development period of expansive soil under different wet and dry cycling conditions were conducted indoors to investigate the change law of shear strength decay. The ring-knife soil samples were humidified with nozzles in order to ensure the same state before each drying. Then the moisture content of the samples was measured with a balance, and their moisture content of was uniformly controlled at 60% before drying. After that, the samples were put into the oven for the same time in order to be fully and uniformly dried. A total of four tests were conducted in each group, two of which were used to compare the fissure development of the specimens under different wet and dry cycles, and the other two groups were used for direct shear tests under different wet and dry cycles. The test results are shown in Figs. 3 and 4. From Fig. 3, it can be seen that the cracking rate of the expansive soil specimens increased at different stages as the number of wet and dry cycles increased. Under the first and second wet and dry cycles, the cracking rate gradually increased slowly, and after the third wet and dry cycle, the cracking rate increased rapidly. At this time, the surface of the soil sample also showed a longitudinal and transverse fracture network, and under the subsequent fourth and fifth wet and dry cycles, the fracture rate changed slowly and the surface fracture network was similar to that after the third wet and dry cycle.

Figure 4 shows the results of the direct shear test of the samples under different dry and wet cycles. Comparing the fracture development in Fig. 3, it can be seen that the soil structure of the expansive soil is destroyed and the shear strength of the soil decreases under the action of dry and wet cycles, among which, the cohesive force decreases significantly with the increase of the number of dry and wet cycles, and the cohesive force decreases to 10.3 kPa after the 8th dry and wet cycle, which decreases by 52.3%. It is worth noting that the stabilized value of cohesive force decay is very close to the cohesive force of 8.8 kPa of the expansive soil at the sliding zone taken from the site borehole, which confirms the reasonableness of the dry and wet cycle simulation scheme adopted in this paper, and also indicates that the expansive soil at the sliding zone of the site has undergone dry and wet cycles, and its cohesive force has decayed to the lowest value, thus causing the sliding damage of the site. It can be seen that the dry and wet cycling is the main reason for the shallow instability damage of the expansive soil slope. On the other hand, the variation of the internal friction angle with the number of wet and dry cycles is not obvious and fluctuates in the range of 11.5° to 11.2°, which is consistent with the experimental results of Huang et al.^25^. Marzulli et al.^26^ and Sandeep et al.^27^ concluded that the internal friction angle depends on the surface roughness and shape characteristics of soil particles, which are inherent properties of the soil. Despite the soil undergoes expansion and contraction, its surface characteristics do not change. The soil undergoes expansion and contraction but its surface characteristics do not change, which can explain the increase in the number of wet and dry cycles with little change in the angle of internal friction.

## Stability analysis of expansive soil slopes

### Expansion force calculation method

**Figure 5:**
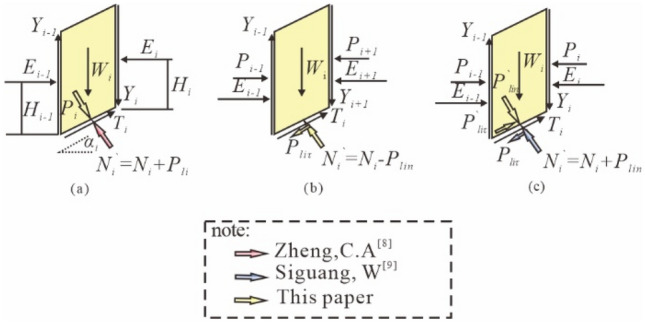
Soil strip model.

When analyzing the stability of swelling soil slopes by the slices method, different scholars have different views on the way the swelling force acts. Yin et al.^28^ regards the swelling force of the soil below the sliding body as a part of the foundation reaction force (i.e., it is regarded as internal force) thus it does not affect the balance of forces on the sliding body. Zheng^29^ consider that the sliding body for the slip cracking surface there is an expansion force *P′*_*li*_, so the sliding surface on the sliding soil will also have a reaction force, that is, the new foundation reaction force *N*_*i*_*′* when the soil swells is the sum of the original foundation reaction force *N*_*i*_ and the reaction force *P*_li_ of the expansion force (Fig. 5a). Si Guangwu et al.^30^ decomposed the expansion force of the sliding body on the slip crack surface into the component force *P′*_*liτ*_ parallel to the slip surface and the component force *P'*_*lin*_ perpendicular to the slip surface. Under the influence of the reaction force, the sliding body will be subjected to the expansion force *P*_*liτ*_ perpendicular to the sliding surface, so the new foundation reaction force *N*_*i*_' is the sum of the original foundation reaction force *N*_*i*_ and *P*_*lin*_ (Fig. 5b). This paper considers that the expansion force belongs to the internal force of the slope, but because the soil below the slip surface also has expansion force reaction to the landslide body. Therefore, the new foundation reaction force *N*_*i*_' should be the difference between the original foundation reaction force *N*_*i*_ and the expansion force *P*_*lin*_, as shown in Fig. 5c.

**Figure 6:**
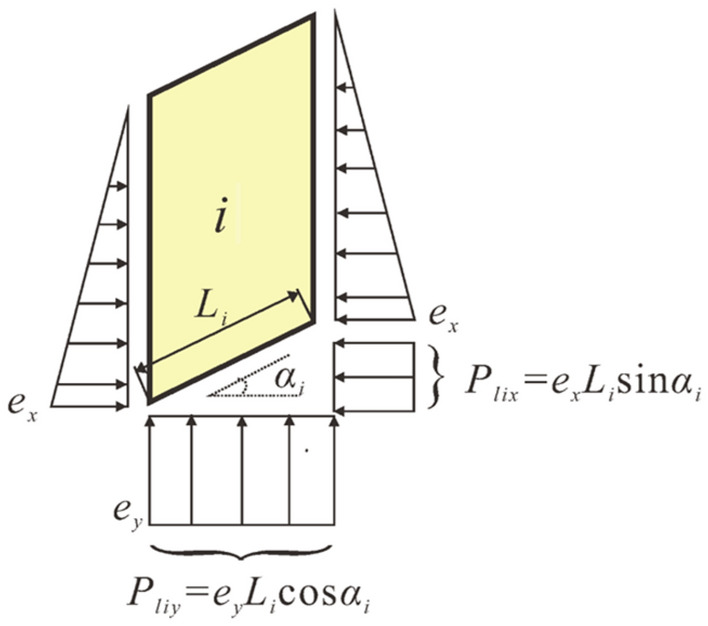
Soil slice swelling force distribution pattern.

As shown in Fig. 5c, take the *i*th slice as an example, the bottom edge of the strip is *L*_i_ long. where *Y*_i-1_, *Y*_i_ are the lateral tangential forces of the slice, *E*_i-1_, *E*_i_ are the normal forces acting on the sides of the slice, *P*_i-1_, *P*_i_ are the lateral expansion forces between adjacent slices, the lateral expansion forces on the slice are assumed to be triangularly distributed, so *E*_i_ and *P*_i_ act at the same location on the slice, *H*_i_ is the vertical distance from the action point of *E*_i_ to the bottom edge of the slice, *W*_i_ is the self-weight of the strip, *N*_i_ is the foundation reaction force on the slip surface, and *N*_i_ is the effective weight of the soil when saturated. *N*_*i*_ is the foundation reaction force on the slip surface of the strip. *T*_*i*_ is the slip resistance force on the slip surface of the strip. *P*_*lit*_ and *P*_*lin*_ are the expansion forces parallel and perpendicular to the slip direction on the slip crack surface of the strip, as shown in Fig. 6, the following relations should be satisfied:

1 $$\begin{aligned} P_{li\tau } & = P_{lix} \cos \alpha_{i} - P_{liy} \sin \alpha_{i} \\ & = L_{i} (e_{x} - e_{y} )\sin \alpha_{i} \cos \alpha_{i} \\ \end{aligned}$$

2 $$\begin{aligned} P_{lin} & = P_{lix} \sin \alpha_{i} + P_{liy} \cos \alpha_{i} \\ & = L_{i} [e_{x} (\sin \alpha_{i} )^{2} + e_{y} (\cos \alpha_{i} )^{2} ] \\ \end{aligned}$$

where *α*_i_ is the inclination angle of the bottom edge of the *i*th slice; *P*_*lix*_ and *P*_liy_ are the horizontal and vertical expansion forces on the bottom of the strip; *e*_x_ and *e*_y_ are the horizontal and vertical expansion force intensity respectively.

### Dry and wet cycle simulation methods

**Figure 7:**
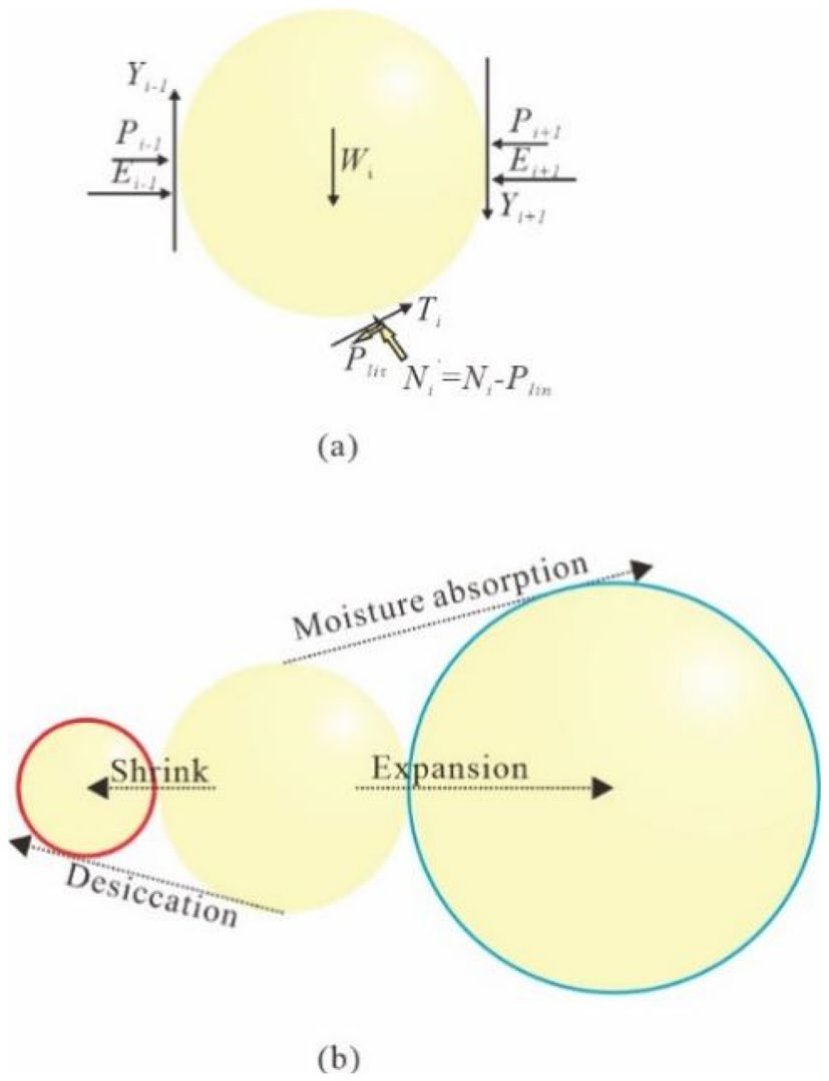
(**a**) Schematic diagram considering expansion forces. (**b**) Expansion and contraction deformation pattern of expansive soil particles.

In DEM simulations, the expansive soil particles can be characterized by spherical particles with bonding between the particles. Due to the large amount of hydrophilic minerals in the expansive clay, it has obvious characteristics of water absorption and expansion and water loss and contraction. Based on the above dry and wet cycle tests, the expansion and contraction mechanism of expansive soil was introduced into the interaction of discrete element clay particles, and a numerical model of water content-deformation was established based on the quantitative relationship between water content and volume change, the simulation schematic is shown in Fig. 7. Referring to the previous settings of the DEM to simulate the volume of soil particles in the dry and wet cycles of clay and related calculation methods, from the microscopic point of view, the expansive soil’s matric suction will increase with the decrease of water content, while the thickness of the water film between soil particles will gradually decrease and the radius of soil particles will also change. Therefore, the mechanical properties of soil particles and the variation of soil particle aggregate should be paid attention to during DEM simulations. The relationship between uniaxial tensile strength and water content of expansive soils and the water-soil characteristic curve can be expressed by the following equation^31^:

3 $$\begin{aligned} \sigma_{{\text{t}}} & = 3729e^{( - w/12)} - 25.21 \hfill \\ \lg \psi &= a + b \cdot \theta_{w} \hfill \\ \end{aligned}$$

where, $\sigma_{{\text{t}}}$ is the uniaxial tensile strength (kPa), *w* is the water content, $\psi$ is the matric suction, a and b are the empirical parameters of the test and equivalent to 6.263 and − 0.1052, respectively.

The pairwise relationship among the bond strength between soil particles, particle stiffness and water content during water loss cracking^31^ is expressed as follows.

4 $$\begin{aligned} T_{n} & = 14.916e^{( - w/12)} - 0.101 \hfill \\ k_{n} & = 1.25 \times 10^{7} e^{( - 0.2w)} \hfill \\ \end{aligned}$$

where *T*_n_ is the interparticle bond strength, *w* the water content and *K*_n_ the interparticle bond stiffness.

The soil volume variation during desiccation cracking is based on the idealized model proposed by Youssoufi et al.^32^. The solid and liquid phases of the soil in the discrete element model are defined as shrinkable clusters during cracking, and the radius of the soil particles in the simulation varies according to the following empirical equation^33^:

5 $$R = R_{0} e^{{\left( { - \beta \frac{N}{{N_{f} }}} \right)}}$$

where *R* is the radius of soil particles, *R*_0_ the radius of N = 0 at the beginning of the test, *β* the soil dry shrinkage coefficient, and *N*_f_ the total duration of the test.

### Material setting and parameter determination

**Figure 8:**
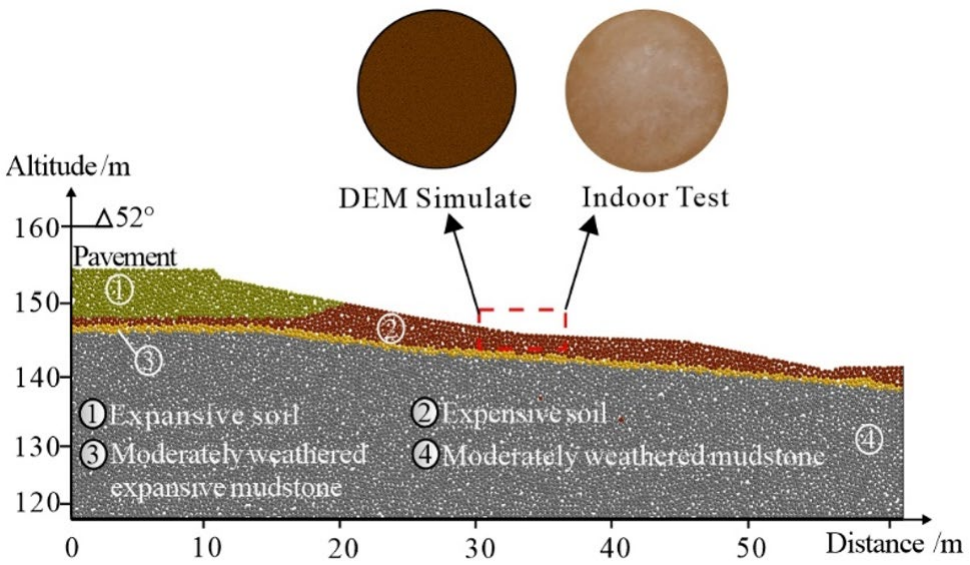
Sketch maps of (**a**) Considering expansion force. (**b**) Particle deformation form.

According to the profile distribution of the embankment slope in Fig. 1, the DEM slope model established is shown in Fig. 8. The mesoscopic mechanical parameters in the discrete element numerical simulation are critical to the accuracy of the simulation. During the simulation, the interparticle contact model was set according to Yoon^34^ et al. for the uniaxial compression test of clay layers under different wet and dry cycles. Based on the uniaxial compression test, the stress–strain curve was used to calibrate the mesoscopic parameters. Since the landslide is a shallow one, and the study area mainly focuses on the upper expansive clay layer, the lower bedrock part does not play a significant role in the stability analysis and its parameters was not calibrated. The calibrated mesoscopic parameters are summarized in Table 2. the mesoscopic mechanical parameters of this DEM simulation were calibrated from two aspects: firstly, ring-knife specimens with the same size as the indoor test were taken from the established DEM slope model for fissure development test under dry and wet cycles; secondly, the stress–strain paths during the unconfined compression of the expanded soil specimens were mechanically calibrated by establishing the same DEM model as the indoor biaxial compression test.

**Table 2:** Numerical simulation of material parameters.

Parameters	Value
Density of particle, *ρ*	Expensive soil: 2750 kg/m^3^
	Miscellaneous soil: 2700 kg/m^3^
Normal stiffness of particle, *K*_*n*_	Expensive soil: 8 × 10^7^ N/m
	Miscellaneous soil: 6 × 10^7^ N/m
Shear stiffness of particle,*K*_*s*_	Expensive soil: 4 × 10^7^ N/m
	Miscellaneous soil: 3 × 10^7^ N/m
Friction coefficient,*μ*_*s*_	Expensive soil: 0.7
	Miscellaneous soil: 0.5
Parallel bond strength, Pb_ten	Expensive soil: 1 × 10^5^N/m
	Miscellaneous soil: 1.5 × 10^4^N/m

**Figure 9:**
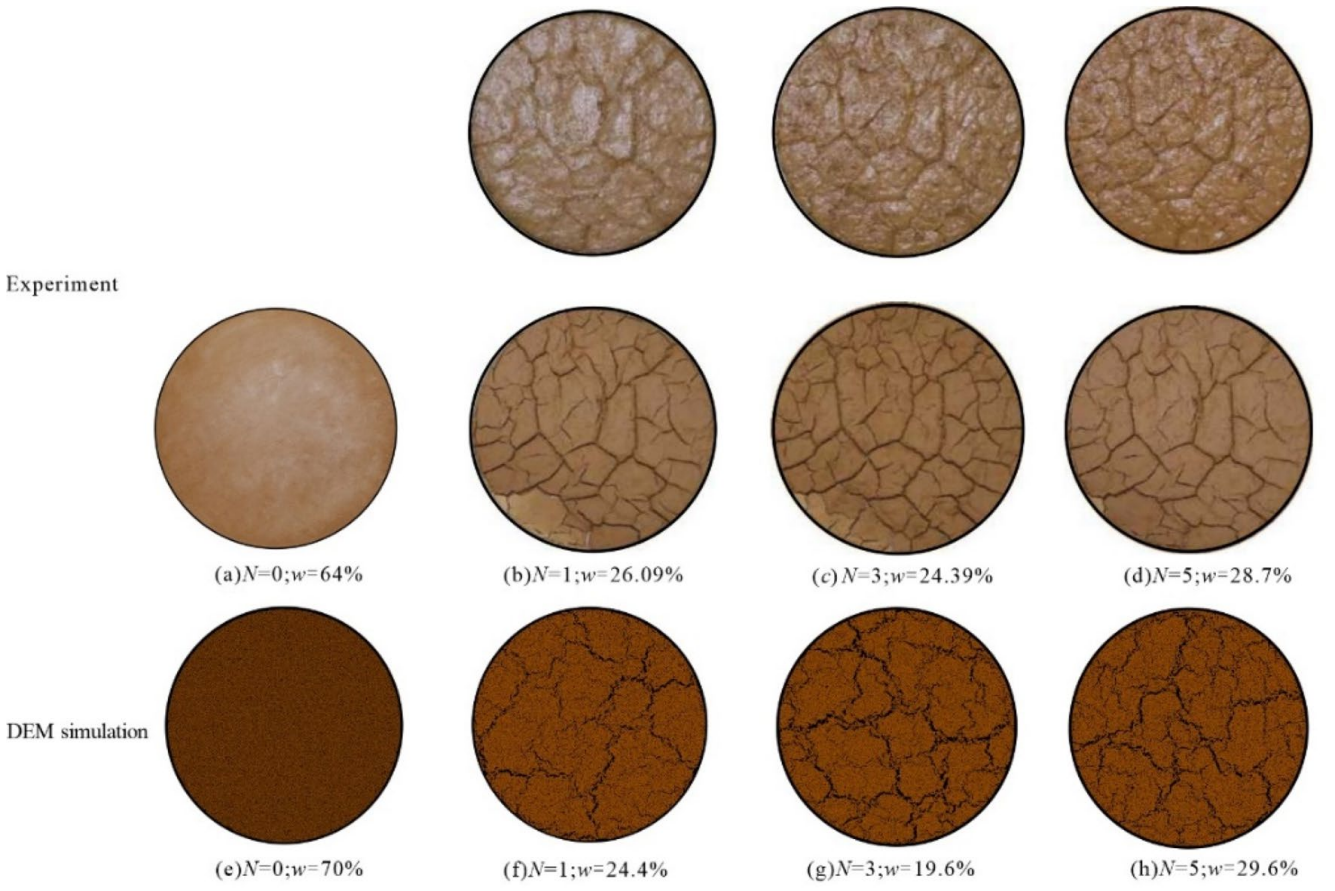
Simulation of dry–wet cycle of expansive soil.

The indoor fissure development test of the expansive soil is shown in Fig. 9. During the simulation, the initial moisture content of the soil sample was set to 70%, the final moisture content was set to 8%. When simulating the dry and wet cycle stages, the following points were considered: the bond and radius change between particles during dry cracking were set with reference to Eqs. (3)–(5); when the fissures developed steadily, the soil humidification stage was simulated, and during the humidification simulation, the radius of particles gradually increased due to the increase of moisture, and the fissures gradually approached the original state; then the further dry shrinkage process was simulated. It can be seen from Fig. 9 that the shape of the fissure development of the ring knife specimen after the 3rd, 4th and 5th dry and wet cycles of the DEM simulation is close to that of the indoor test, dynamically reproducing the fissure development of the soil sample under different dry and wet cycles.

**Figure 10:**
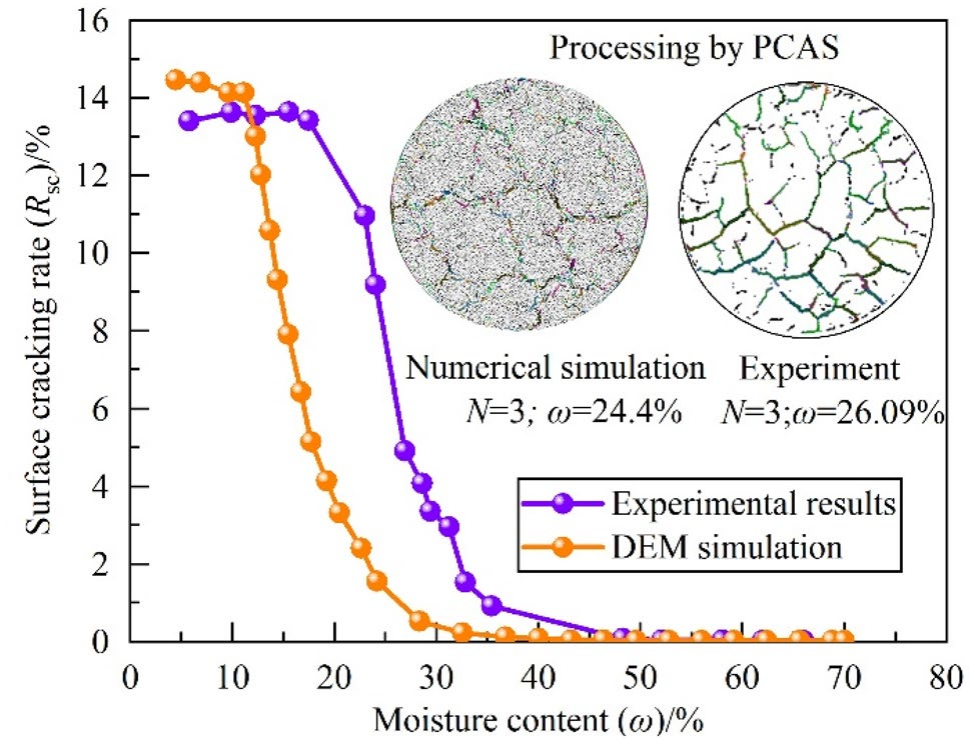
Simulation of dry–wet cycle of expansive soil.

In order to quantitatively reflect the fissure development of the DEM simulation on the desiccation process of the expansive soil samples, the image processing software PCAS developed by Liu^35^ was used to describe the fissure development at different moisture contents of the samples obtained from indoor tests and numerical simulations, taking the desiccation stage in the third dry–wet cycle as an example. The comparative curves of numerical simulation and indoor test for the fissure rate-water content change are shown in Fig. 10, from which it can be seen that the DEM results is relatively close to the results of the indoor test, both of which indicate that the fissure rate of the specimen goes through a "stable→growth→faster growth→stabilization" stage during the drying process. "The DEM and the indoor model test results are close to each other. Although there are some gaps in the overlapping curve fitting, it can generally reflect the desiccation fissure evolution characteristics of the expansive soil samples under the action of dry and wet cycles.

**Figure 11:**
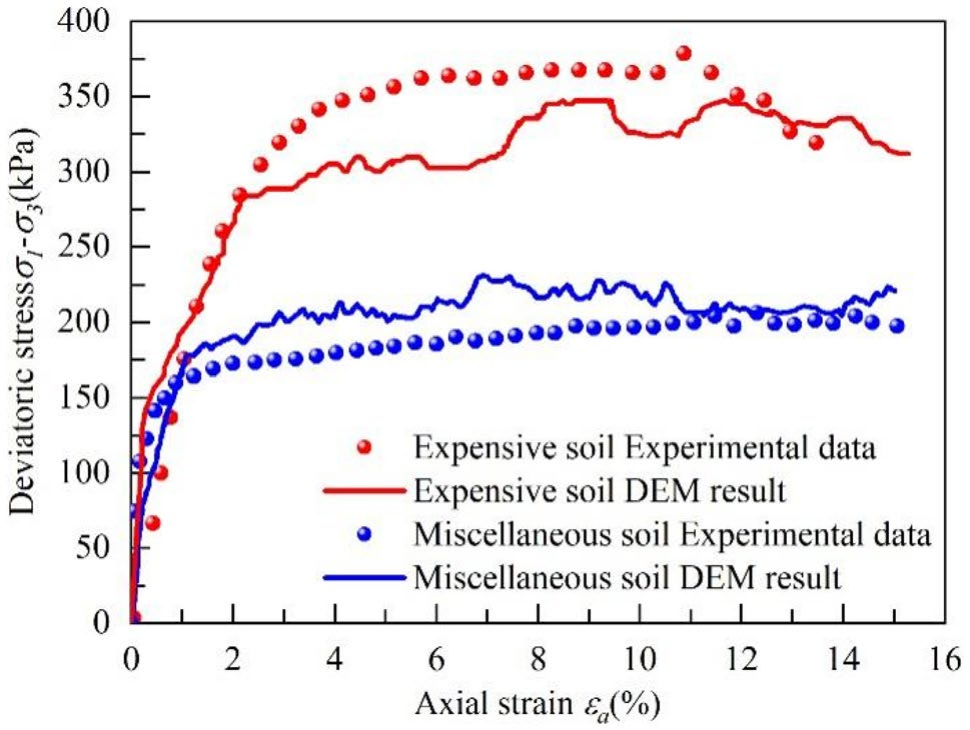
Mechanical parameter calibration result.

In the calibration process of the mesoscopic parameters, the numerical model was established by referring to the indoor biaxial model test, which contains 23,240 soil particles. The calibration results are shown in Fig. 11, which shows that the numerical simulation results of the "stress–strain" curve are close to that of the indoor biaxial experiment. Although there are some differences in the values, which was caused by the differences in the simulation dimensions, the physical and mechanical properties of the key soil layer of the landslide can be characterized to a certain extent from the microscopic perspective.

### DEM results

**Figure 12:**
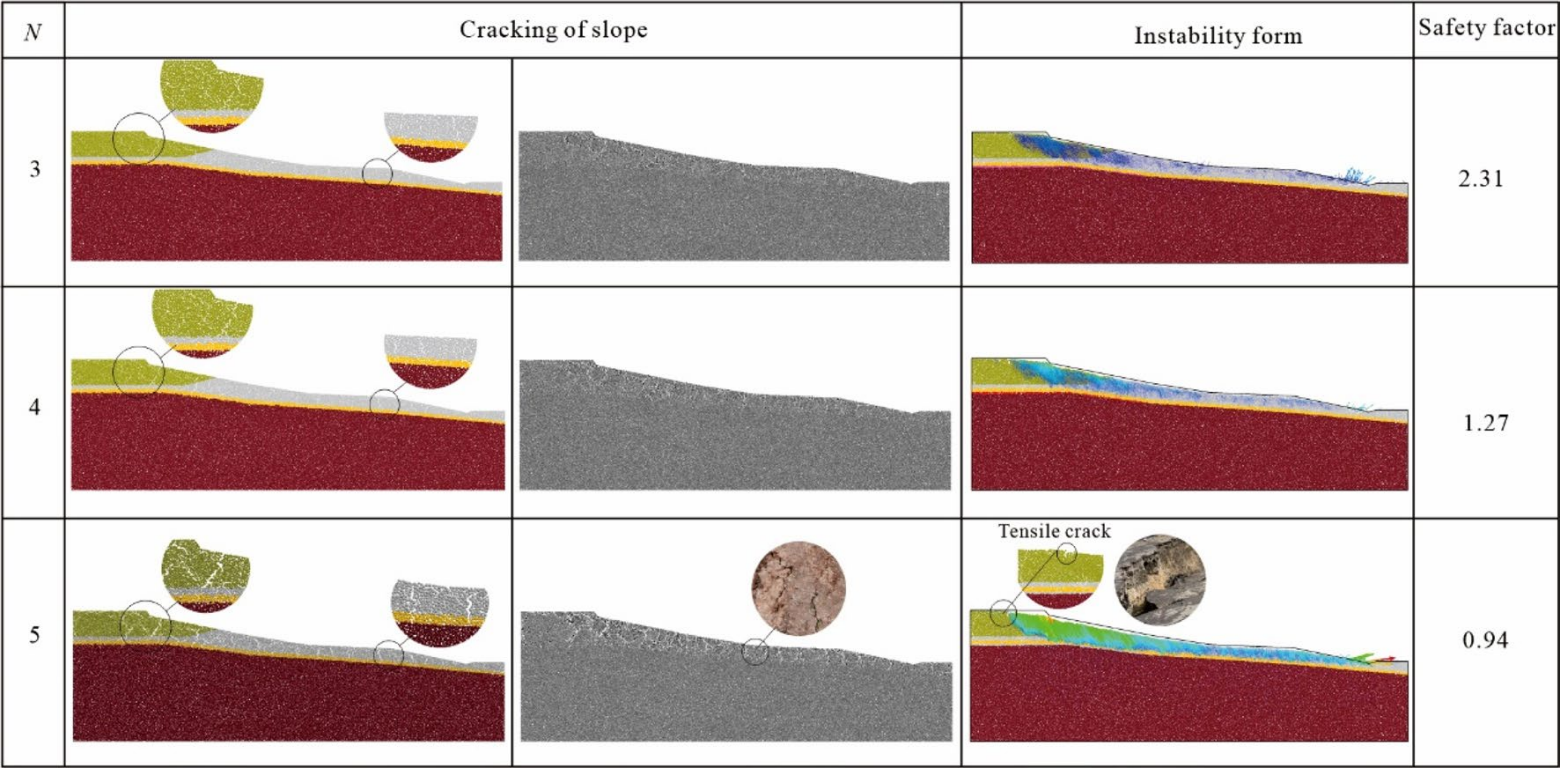
Mechanical parameter calibration results.

Based on the above calculation method of swelling force, the relationship between the deformation of expansive soil particles and water content under the action of dry and wet cycles, the simulation of fissure development of expansive soil ring knife specimens under the action of dry and wet cycles and the calibration results of mesoscopic physical and mechanical properties of sensitive soil layers, the simulation of the sliding of embankment slope of expansive soil in Ningming, Guangxi was carried out, and the simulation results are shown in Fig. 12. The safety factor has been used in this work to evaluate the state of the expansive slope, generally, the safety factor is defined as the ratio of the sliding resistance force to the slipping force in assuming a slipe surface, and different safety factors corresponding to the different slope status^28–30^, as shown in Table 3.

**Table 3:** Potential hazard degree of the slope corresponding to different safety factors.

Safety factor (Fs)	Fs < 1	1 < Fs < 1.05	Fs > 1.05
Slope state	Instable	Limit state	Stable

The development degree of slope fissures, change of safety coefficient and sliding under different dry and wet cycle conditions can be obtained by gravity increase method on the established DEM model. It can be seen from Fig. 12 that the safety coefficient was 2.31 when the number of dry and wet cycles of the swelling soil slope reached 3. At this time, fissures were generated in a certain range on the slope surface, but the slope was in a stable state in general. It can be seen from Fig. 12 that the disturbance range was concentrated in the arc range from the leading edge of the embankment to the foot of the slope. When the number of wet and dry cycles of the slope reached 4, the safety coefficient dropped to 1.27. At this time, the cracks on the surface of the slope were densely developed, and the soil at the leading edge of the slope top sank and exhibited an obvious sliding trend. From the displacement pattern, it can be seen that the sliding surface tended to be shallowly circular from the leading edge of the embankment to the foot of the slope. When the number of wet and dry cycles reached 5, the surface connection of the slope became obvious, the fissures were interconnected and extended to the deep part of the slope, and the safety coefficient dropped to 0.94. The sliding surface shape of the swelling soil slope was obvious, and the slope was in an unstable state. From the displacement vector distribution of the slope, it can be seen that the sliding surface of the slope cut from the front edge of the embankment to the foot of the slope, and the displacement gradually decreased from the front edge of the embankment to the gully at the foot of the slope. The sliding form of the embankment slope and the tensile fissures on the slope were similar to what were observed in the field investigation. From the numerical simulation results, it can be seen that the DEM simulation can well reproduce the fissure development and the destabilization evolution process of the expansive soil slope under the action of dry and wet cycles after introducing the expansion force and calibrating the fissure development form and micromechanical properties of the soil body under the dry and wet cycles.

**Figure 13:**
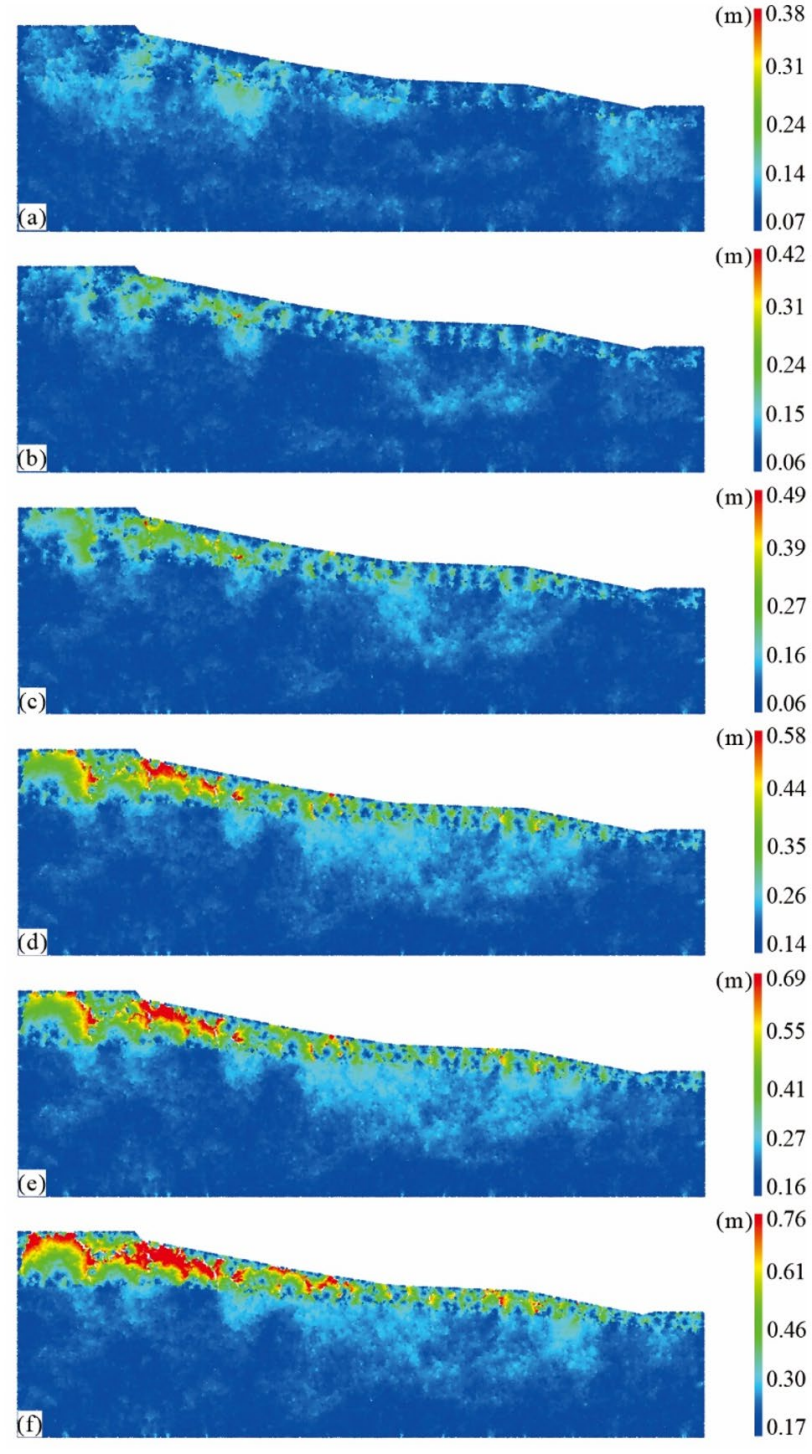
Evolution of dry destabilization of expansive soil slopes after the fifth wet and dry cycle.

In order to further analyze the cracking evolution process and destabilization mechanism of expansive soil slopes, the stability change of expansive soil slopes under the fifth wet and dry cycle is taken as an example to explore the fissure development and slip zone evolution process of the slopes. The simulation results are shown in Fig. 13, which divides the process of slope cracking and destabilization into six stages, with a total calculated time step of about 1,275,400. From Fig. 13, it can be seen that when the slope of expansive soil after five dry and wet cycles underwent desiccation, cracks started to appear gradually on the upper surface of the slope, which are concentrated in the pavement area at the top of the slope, and the development of cracks started from the shallow interior of the slope, which may be caused by the uneven shrinkage direction of the soil in the process of drying and shrinking, and the relatively rapid shrinkage behavior of the soil on the slope surface. The fissures gradually extended to the deeper part as the drying of the swelling soil slope continued. From Fig. 13b and c, it can be seen that the fissures in the shallow slope body were most densely developed at the leading edge of the slope top and affected the widest depth range. With the continuous development of the fissures, the fissures in the front edge of the slope body and in the interior of the shallow slope body gradually started to penetrate. From Fig. 13d, it can be seen that some fissures on the leading edge of the slope have penetrated each other, extending from the middle of the roadway to the leading-edge slope. From Fig. 13e and f, it can be seen that the fissures in the slope were obviously connected with each other, showing an obvious trend of penetration. The penetration area covered the front edge, the middle and the foot gully of the slope, and the evolution of the slip zone was obvious.

### Slope stability analysis

**Figure 14:**
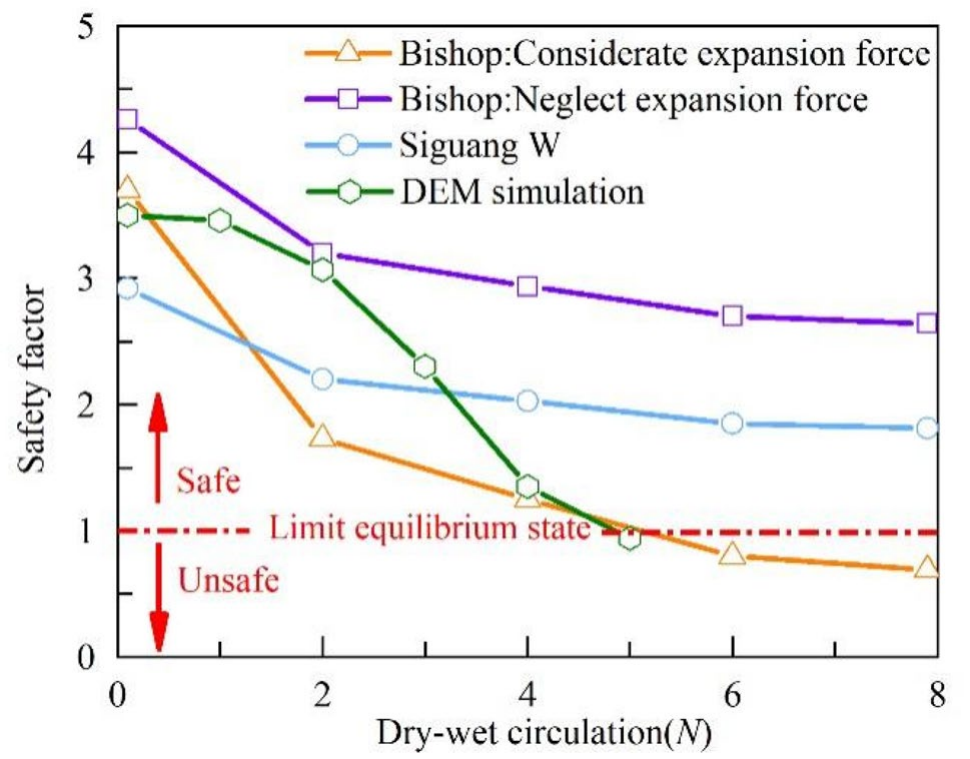
Coefficient of safety of swelling soil slopes obtained by different methods under the action of dry and wet cycles.

In order to compare and verify the accuracy of introducing the swelling force and dry and wet cycle fissure evolution methods into the DEM simulation in this paper, the expansive soil landslide in Ningming, Guangxi, was used as an engineering example, and the results of the swelling force and shear strength tests under the above dry and wet cycles were divided into 15 slices according to the aforementioned Janbu method to calculate the stability change of this swelling soil slope under the action of dry and wet cycles, and the lateral expansion force was taken as 25% of the vertical expansion force, i.e., 10.2 kPa^36^. Figure 14 shows the variation of the slope safety factor with the number of dry and wet cycles obtained by the DEM simulation, the Janbu method with the introduction of swelling force, the method proposed by Si Guangwu et al.^30^, and the method without considering swelling force^29^.

From Fig. 14, it can be seen that the introduction of expansion force reduced the slope safety factor before being affected by several wet and dry cycles, but it was not enough to make the slope unstable. With the increase of the number of wet and dry cycles, the slope safety coefficient showed a trend of rapid decrease and then a slow decrease, which was consistent with the trend of soil shear strength with the number of wet and dry cycles. When the influence of swelling force was not considered, the slope safety coefficient obtained from 8 wet and dry cycles was 2.39, which was much larger than 1. This is contrary to the sliding instability of the actual slope project, indicating that the influence of swelling force on the shallow damage of swelling soil slope cannot be ignored.

After 8 dry and wet cycles, the slope safety factor obtained by Si Guangwu et al. was 1.61, and the slope was still in a stable state, which is not consistent with the actual slope state. The slope safety factor obtained by the DEM simulation and the Janbu method shows a conspicuous change trend with the increase of the number of wet and dry cycles. After 5 dry and wet cycles, the slope safety factor obtained by the DEM simulation was 0.94, and the slope safety factor obtained by the Janbu method was 1.08. At this time, the slope became unstable, which is consistent with the actual destabilization damage state of the slope. It can be seen that the proposed method takes into account the effect of swelling force and dry and wet cycles, and reasonably deduces the change process of gradually decreasing stability of the slope of swelling soil under the joint action of dry and wet cycles and swelling force. It should be pointed out that due to the special characteristics of expansive soil, its slope destabilization damage mechanism is more complicated than that of common soil slopes. Therefore, the discrete element method and the Janbu method introduced in this paper provide important references for the stability analysis of similar expansive slopes. However, since the analysis belongs to two-dimensional calculation mode and has some dimensional difference from three-dimensional reality, it needs further verification and improvement in the future.

## Conclusions

In this paper, a discrete element numerical simulation method of expansive soil slope considering the influence of swelling force and dry and wet cycles of swelling soil is proposed to study the process of fissure evolution and stability of expansive soil slope under the action of dry and wet cycles, taking the sliding of expansive soil embankment of a national highway in Ningming, Guangxi as a case. In the DEM model, in addition to the contact force, the volume change of particles due to the change of water content and the consequent change of swelling force are also considered. By establishing the relationships among water content and particle radius, Young's modulus, tensile strength and swelling force, the influence of the relationship between moisture and stress of expansive soil on the fissure evolution and stability of expansive soil slope is considered comprehensively. The indoor wet and dry cycle fracture expansion test and biaxial compression test verified the objectivity of the proposed DEM model; and its reliability was verified in the simulation of the sliding of the expansive soil embankment. The accuracy of the DEM was verified by comparing with Janbu method, which considers swelling force and wet and dry cycles, and bishop method, which considers swelling force but not wet and dry cycles. Based on the proposed DEM model established by considering swelling force and dry and wet cycles, the following conclusions can be drawn:

1. The physical and mechanical properties of expansive soil gradually decrease with the increase of the number of dry and wet cycles, the cohesive force between soil particles gradually decreases under multiple dry and wet cycles.
2. During the desiccation process, the volume of soil shrinks and soil particles move to the shrinkage center distributed in different spaces of the slope body, and, consequently, the fissures are generated.
3. Under the action of dry and wet cycles, the starting point of fissure development may be in the slope rather than at the surface, due to the shrinkage behavior of the soil body. As the number of dry and wet cycles increases, the fissures developed in the deep part of the shallow slope gradually penetrate each other, forming an obvious fissure sliding surface and prompting the shallow slope to stagger along the fissure sliding surface.
4. Based on the numerical simulation results, it can be found that the failure of an expansive soil slope has a typical shallow and step-by-step nature, so the replacement of shallow expansive soil or the installation of a retaining wall at the slope foot can effectively alleviate the destabilization damage caused by dry and wet cycles compared to other measures.

## Data Availability

All data, models, or code generated or used during the study are available from the corresponding author by request.
